# Dietary Manganese Modulates Microbiota and Intestinal *N*‐Acylethanolamines in a Sex‐Specific Manner in Mice With Diet‐Induced Obesity

**DOI:** 10.1096/fj.202500934R

**Published:** 2025-06-26

**Authors:** Fredy Alexander Guevara Agudelo, Nadine Leblanc, Isabelle Bourdeau‐Julien, Gabrielle St‐Arnaud, Fadil Dahhani, Nicolas Flamand, Alain Veilleux, Vincenzo Di Marzo, Frédéric Raymond

**Affiliations:** ^1^ Centre Nutrition, santé et société (NUTRISS), and Institute of Nutrition and Functional Foods (INAF) Université Laval Québec City Québec Canada; ^2^ Canada Excellence Research Chair on the Microbiome – Endocannabinoidome Axis in Metabolic Health (CERC‐MEND) Université Laval Québec City Québec Canada; ^3^ Faculty of Agriculture and Food Sciences Université Laval Québec City Québec Canada; ^4^ Faculty of Medicine Institut Universitaire de Cardiologie et Pneumologie de Quebec, Université Laval Québec City Québec Canada

**Keywords:** endocannabinoid system (eCB), inflammation, intestine, manganese, metabolic health, microbiome, nutrition

## Abstract

Obesity is characterized by low‐grade inflammation, changes in gut microbiota, and increased tone of the expanded endocannabinoid system (eCBome). The gut is a complex ecosystem that serves as an interface between the diet and its ultimate conversion to nutrients and energy for all cells of the organism. Manganese (Mn) is an essential micronutrient from the diet required for normal cell function and physiological processes. Moreover, it is an indispensable trace mineral for some microbial species since it is a cofactor in several metabolic enzymes. Therefore, we aimed to identify possible interactions between gut microbiota composition and the eCBome during dietary variations of Mn in the context of diet‐induced obesity. Female and male mice were fed Mn‐depleted and Mn‐enriched diets in combination with Low Fat‐Low Sucrose (LFLS) or High Fat‐High Sucrose (HFHS) diets for 28 days to assess intestinal microbiota and eCBome levels. Mn‐enriched diets enhanced the circulating levels of *N*‐docosapentaenoyl‐ethanolamine (DPEA) and anandamide (AEA), while they reduced the intestinal concentrations of other *N*‐acylethanolamines, particularly in the caecum. Besides, we found a strong sex effect of Mn on the intestinal levels of 2‐monoacylglycerols (2‐MAGs), which were lower in females. Other endocannabinoid‐like molecules involved in the immune response were impacted by dietary Mn enrichment, such as *N*‐palmitoyl‐glycine and *N*‐oleoyl‐L‐serine. Concomitantly, Mn enrichment promoted segment‐specific changes in the relative abundance of several taxa in intestinal microbiota following the HFHS diet. Microbial families such as *Peptostreptococcaceae*, *Muribaculaceae*, and *Erysipelotrichaceae* responded differentially to dietary variations in Mn. This study hints at potential interactions between Mn levels and diet composition with the eCBome and intestinal bacteria during dietary Mn variations within HFHS‐induced dysmetabolic processes in a sex‐dependent manner. These results will eventually contribute to identifying members of the gut microbiome and mediators of the eCBome useful for improving metabolic health.

## Graphical abstract

Female and male mice were fed manganese (Mn)‐depleted or Mn‐enriched diets in combination with either a low fat‐low sucrose (LFLS) or high fat‐high sucrose (HFHS) diet for 28 days. A sex‐specific metabolic response was observed under Mn‐depleted conditions, particularly affecting monoacylglycerol levels. In contrast, Mn‐enriched conditions revealed diet‐dependent differences in plasma *N*‐acylethanolamines and gut microbiome composition between HFHS and LFLS diets. These findings support the hypothesis that the metabolic effects of micronutrients like manganese are modulated by both dietary composition and sex.

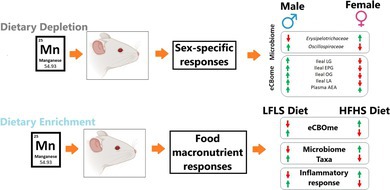

## 1 Introduction

Manganese (Mn) is an essential trace element in multiple mammalian and bacterial physiological processes [1], and a component or activator of several enzymes (oxidoreductases, transferases, hydrolases, lyases, isomerases, and ligases). The activation of these enzymes contributes to the reduction of oxidative stress against free radicals. Mn also plays an important role in carbohydrate and lipid metabolism, vitamin C and vitamin B synthesis, and the regulation of the endocrine system and immune function [2]. Therefore, Mn may have protective effects on the occurrence of the metabolic syndrome [3]. While there are only a few studies on Mn and lipids, a recent report showed that exposure to low‐level Mn reduced serum triglyceride levels in rats [4]. Mn‐induced damage at the tissue level may involve multiple mechanisms, including oxidative stress [5], activation of pro‐inflammatory mediators, neuroinflammation [6], and alterations of ionic homeostasis [7]. On the other hand, it has been reported that Mn‐deficiency impairs normal growth, reproductive function, insulin sensitivity, lipid metabolism, and reduces blood HDL cholesterol [2], which might relate variations of the levels of this mineral to the occurrence of inflammation. In addition, Mn intake has been shown to induce beneficial effects in reversing metabolic dysfunction, including obesity. Conversely, higher Mn intake (e.g., > 5.12 mg/day) was associated with hypertriglyceridemia among men in China [8], and high‐density lipoprotein cholesterol (HDL‐C) levels in women increase with the intake of this mineral. Besides, it has been established that plasma Mn concentrations were significantly higher in obese men aged 50–75 years [9], which again suggests the influence of this mineral in the development of metabolic syndromes.

The expanded endocannabinoid system, or endocannabinoidome (eCBome), is a diverse biological group of lipid mediators including the endocannabinoids, *N*‐arachidonoyl‐ethanolamine (anandamide, AEA), and 2‐arachidonoyl‐glycerol (2‐AG), which mostly bind to two G protein‐coupled receptors, the type 1 (CB_1_), and type 2 cannabinoid receptors (CB_2_), expressed throughout the body [10]. The congeners of AEA and 2‐AG, the *N*‐acylethanolamines (NAEs) and 2‐monoacylglycerols (2‐MAGs), respectively, along with other long‐chain fatty acid amides, are important components of the eCBome and are implicated in several biological processes essential for metabolic health [11]. The eCBome plays a key role in food intake and energy metabolism, satiety, and metabolic control, implying that its imbalance increases the risk of developing the metabolic syndrome, including obesity [12, 13, 14]. Food intake and energy storage increase due to stimulation of CB_1_ receptors by AEA and 2‐AG [15]. Other eCBome mediators, instead, inhibit food intake, such as *N*‐oleoyl‐ethanolamide (OEA), while others, such as *N*‐palmitoyl‐ethanolamide (PEA), exhibit anti‐inflammatory activity [16].

The crosstalk between the intestinal eCBome and gut microbiota regulates many gastrointestinal functions, such as nutrient absorption and immune response [17]. In addition, along with the implications of the eCBome in the development of obesity, it has also been shown that the gut microbiome also plays a preponderant role in the development of metabolic syndromes [18]. Indeed, several studies refer to changes in microbial populations as being highly correlated with the development of obesity [19]. Recent studies have shown that trace minerals can modify the intestinal microbiota [20, 21], suggesting the possible modulatory role that Mn could have on microbial populations. Indeed, several intestinal bacteria, and pathogens such as certain *Yersinia* spp. and *Salmonella enterica Typhimurium* have demonstrated the ability to transport, metabolize and utilize Mn to survive [22, 23]. Interestingly, the intestinal microbiome may compete with the host for the Mn to persist in the gut, but the host has evolved mechanisms to sequester Mn and prevent bacterial access to it [24]. Although there are several impacts of macronutrients on the gut microbiome, there are significantly fewer studies investigating the effects of the exposure of micronutrients and their ability to alter the gut microbiome. In addition, sex‐dependent effects of Mn exposure on the gut microbiome have been described [25], and similarly, endocannabinoid signaling has also been observed to often occur in a sex‐dependent manner [26].

Considering the role of Mn as a cofactor of several enzymes associated with inflammation and its implication with the peroxidation of phospholipids, the ultimate biosynthetic precursors of endocannabinoids, the aim of the present work was to determine how the dietary variations in this mineral affect the host production of eCBome mediators, and if intestinal microbial populations, in interaction with the diet macronutrient composition, respond to these dietary variations, as previous studies have demonstrated the impact of Mn on intestinal microbial communities [25]. In this work, we investigated howMn‐depleted (0.1 mg/kg) or enriched (20 mg/kg) diets—modulate the eCBome and gut microbiome response to a Low Fat‐Low Sucrose (LFLS) or High Fat‐High Sucrose (HFHS) diet in an obesity mice model. Intake of Mn was chosen to represent the full range of safe dietary intake, including low and high Mn consumption without inducing deficiency nor toxicity. We hypothesized that variations in dietary Mn concentrations would affect the concentrations of eCBome mediators at the circulatory and intestinal levels, and their possible interaction with the host microbiome. Thus, experiments were performed in mice (male and female) to examine the impact of Mn and diet composition on these two complex systems. The data were then stratified to assess the influence of sex on the responses of the mice to Mn.

## 2 Materials and Methods

### 2.1 Animals, Diets, and Housing

The study was approved by the Université Laval animal ethics committee (CPAUL 2019‐006). Forty‐eight 6‐week‐old C57BL/6J male and female mice were purchased from Jackson Laboratory (USA) and were individually housed in the animal facility of the Institute of Nutrition and Functional Foods (INAF), in standard cages under controlled temperature (22°C) and relative humidity (50%) with a 12 h day/night cycle. At arrival, all mice were acclimated to their new environment for a 1‐week adaptation period, during which they received a normal chow diet (AIN‐93G‐purified diet #110700, Dyets Inc., Bethlehem, PA, USA). Following this time, they were randomly assigned to 4 groups (*n* = 12 per group, 6 males and 6 females). The groups were defined according to 4 diet designs as follows: Enriched (20 mg/kg) and depleted (0.1 mg/kg) concentrations of Mn in combination with High Fat‐High Sucrose (HFHS: 23.6% fat, 17% sucrose, Research Diets Inc., NJ, USA), and Low Fat‐Low Sucrose (LFLS: 4.3% fat, 7% sucrose, Research Diets Inc., NJ, USA). This sample size has been sufficient in previous studies to detect biologically significant differences [21, 27] while complying with ethical guidelines promoting the reduction of animal use. In this study, Mn was provided as manganese carbonate. The diets were formulated to be isoenergetic, isolipidic and isonitrogenous. Total energy in diets was determined with an adiabatic Parr 6300 calorimeter (Parr Instrument Company, Moline, IL, USA) and was different among the four diets (Mn‐depleted LFLS 3952.85 cal/g; Mn‐enriched LFLS 3881.65 cal/g; Mn‐depleted HFHS 4868.6 cal/g; Mn‐enriched HFHS 4994.8 cal/g). Dietary protein content was determined by combustion (Dumas method) using a LECO FP‐528 apparatus (LECO Corporation, St. Joseph, MI, USA) and was found similar between the diets (Mn‐depleted LFLS 14.73% [w/w]; Mn‐enriched LFLS 14.70% [w/w]; Mn‐depleted HFHS 18.09% [w/w]; Mn‐enriched HFHS 18.99% [w/w]). Dietary fat content was measured with an ANKOMXT10 Extractor (ANKOM Technology, Macedon, NY, USA) and was different between the diets, reflecting the fact that we have low‐fat and high‐fat diet (Mn‐depleted LFLS 2.74% [w/w]; Mn‐enriched LFLS 3.27% [w/w]; Mn‐depleted HFHS 22.46% [w/w]; Mn‐enriched HFHS 21.73% [w/w]). Animals were fed *ad libitum* with these diets for 28 days and had access to *ad libitum* water. Body weight and food intake were monitored twice weekly. Mice were killed by cardiac puncture after anesthesia. Whole blood was collected in K3‐EDTA tubes to obtain plasma (1780 × g, 10 min). Ileum and caecum tissues were collected at 10 and 2 cm from the ileocecal junction, respectively. Luminal contents were collected in PBS by gentle scraping. Tissue samples from both ileum and caecum were treated with RNAlater Stabilization Solution (ThermoFisher, USA) to preserve the integrity of RNA until its subsequent extraction. All samples were stored at −80°C until further analysis.

### 2.2 Endocannabinoidome Quantification

Lipids were extracted from plasma samples (40 μL) as in [28]. In brief, plasma samples were diluted to a volume of 500 μL with Tris buffer (50 mM, pH = 7). 5 μL of deuterated standards were added to each sample, then vortexed. Two milliliters of toluene were then added, and samples were vortexed for 30 s. Samples were next placed in a dry ice‐ethanol bath to freeze the aqueous phase. The toluene phase was then collected and evaporated to dryness under a stream of nitrogen. Ileum and caecum samples (5–10 mg) were extracted and processed exactly as in [29]. All lipid extracts were then resuspended with 60 μL of mobile phases (50% Solvent A and 50% solvent B) then injected (40 μL) onto an HPLC column (Kinetex C8, 150 × 2.1 mm, 2. 6 μM; Phenomenex) as described before [30]. Quantification of eCBome‐related mediators was performed using a Shimadzu 8050 triple quadrupole mass spectrometer. The following metabolites were quantified: 1/2‐oleoyl‐glycerol (2‐OG), 1/2‐linoleoyl‐glycerol (LG), 1/2‐arachidonoylglycerol (2‐AG), 1/2‐eicosapentaenoyl‐glycerol (2‐EPG), 1/2‐docosapentaenoyl(n‐3)‐glycerol (2‐DPG), 2‐docosahexaenoylglycerol (1/2‐DHG), *N*‐palmitoyl‐ethanolamine (PEA), *N*‐stearoylethanolamine (SEA), *N*‐oleoyl‐ethanolamine (OEA), *N*‐linoleoyl‐ethanolamine (LEA), *N*‐arachidonoyl‐ethanolamine (AEA), *N*‐eicosapentaenoyl‐ethanolamine (EPEA), *N*‐docosapentaenoyl‐ethanolamine (DPEA), *N*‐docosahexaenoyl‐ethanolamine (DHEA), arachidonic acid (AA), docosahexaenoic acid (DHA), docosaepentaenoic acid (DPA), eicosapentaenoic acid (EPA), stearidonic acid (SDA), linoleic acid (LA), prostaglandin D2 (PGD_2_), prostaglandin E1 (PGE_1_), prostaglandin E1 (PGE_2_), prostaglandin E3 (PGE_3_), 1a,1b‐dihomo Prostaglandin PGF_2α_ (1a,1b‐dihomo PGF_2α_), thromboxane B_2_ (TBX), *N*‐Palmitoyl‐Glycine, and *N*‐Oleoyl‐Serotonin. For the MAGs, the signals from the *sn*‐1 [3] and the *sn*‐2 isomers were combined and presented as 2‐MAGs to take into account the rapid isomerization of the *sn*‐2 isomer to *sn*‐1 [3]. For the endocannabinoidome mediators' analyses, 2 mice were excluded for caecum as they were considered outliers using PCA; thus, the number of mice distributed for the analyses was as follows: *n* = 12 for plasma, *n* = 12 for ileum, and *n* = 10 for caecum.

### 2.3 16S rRNA Gene Sequencing

Intestinal luminal contents were lysed using bead beating (0.1 mm silica beads) before enzymatic digestion with 50 mg of lysozyme and 200 U of mutanolysin by using 10 μL of a solution at 20 U/μL mutanolysin (37°C, 45 min). Microbial DNA was extracted using the QIAamp DNA Stool mini kit (Qiagen, CA, USA), and amplification of the V3‐V4 region was performed using the primers Bact‐0341‐b‐S‐17 (5′‐CCTACGGGNGGCWGCAG‐3′) and Bact‐0785‐a‐A‐21 (5′‐GACTACHVGGGTATCTAATCC‐3′) (Illumina, CA, USA). Libraries were purified using magnetic beads AMPURE XP (Beckman Coulter Canada Lp), and libraries were assessed on gel using QIAexcel (Qiagen, CA, USA). High‐throughput sequencing (2‐ by 300‐bp paired end) was performed on a MiSeq platform (Illumina, CA, USA). Sequences were processed using the DADA2 package (version 1.16.0) [31] and associations with bacterial taxa were obtained using the Ribosomal Database Project reference database Silva version 132. Microbiome abundances were normalized using rarefaction (Rarefaction; Vegan R package). Reads were rarefied to 5000 reads to account for depth bias [32]. Samples with read counts lower than 5000 but higher than 2000 reads were kept in the analysis as is. Prior to rarefaction, we observed 5113 ASV and after rarefaction we observed 4923 ASV. Two males (Mn‐depleted HFHS and Mn‐Enriched LFLS) and one female (Mn‐enriched HFHS) mouse were removed as they had low numbers of sequences. Raw sequences were deposited to SRA under accession PRJNA1005664.

### 2.4 mRNA Isolation, Reverse Transcription, and qPCR

RNA was extracted from the ileum and caecum samples with the RNeasy Plus mini kit (Qiagen, CA, USA) according to the manufacturer's instructions and eluted in 30 μL of UltraPure distilled water (Invitrogen, USA). RNA concentration and purity were determined by measuring the absorbance of RNA in a nanodrop at 260 and 280 nm. A total of 500 nanograms of RNA was reverse transcribed with a high‐capacity cDNA reverse transcription kit (Applied Biosystems, CA, USA). We used the 7500 Real‐Time PCR System (Applied biosystems, CA, USA) to perform quantitative PCR to assess the expression of 4 eukaryotic genes related to inflammation (*Il10*, *Tgfb1*, *Il1b*, and *Tnfa*) with one housekeeping gene (*Hprt*). Primers and probes for TaqMan qPCR assays were purchased as commercial kits (ThermoFisher Scientific, Burlington, ON, Canada) and TaqMan assay IDs were as follows: *Hprt* (Mm03024075_m1), *Il10* (Mm01288386_m1), *Tgfb1* (Mm01178820_m1), *Il1b* (Mm00434228_m1), and *Tnfa* (Mm00443258_m1). All expression data were normalized by the threshold cycle (2^−ΔΔCT^) method using *Hprt* as the internal control [33].

### 2.5 Statistical Analyses

Data are expressed as mean ± SEM. Generalized linear regression models were used to identify the effects of Mn, diet and sex on ranked values of eCBome mediators and gut microbiome relative abundances. We used a three‐way ANOVA based on a linear model that included interactions between diet formulation (LFLS vs. HFHS), Mn concentration (depleted vs. enriched) and sex of the animal (female or male). The differences were considered statistically significant with *p* values of *p* < 0.05 using contrast test between Mn‐depleted and Mn‐enriched levels, LFLS and HFHS formulations, the sex of animals (female and male) and the combination between Mn levels and diet formulations. Spearman correlations were used to investigate associations between microbiome families and eCBome mediators and *p*‐values were adjusted for multiple testing using false‐discovery rate (FDR). Analyses were performed with R software version 4.0.2. Principal‐component analysis was performed using the FactoMineR R package [34]. PERMANOVA was performed between two of the segments of intestine (ileum and caecum) with 999 permutations in conjunction with Canberra distances between samples using package vegan in R (v2.5.7).

## 3 Results

### 3.1 Dietary Manganese Intake Has No Impact on Weight Gain

**FIGURE 1:**
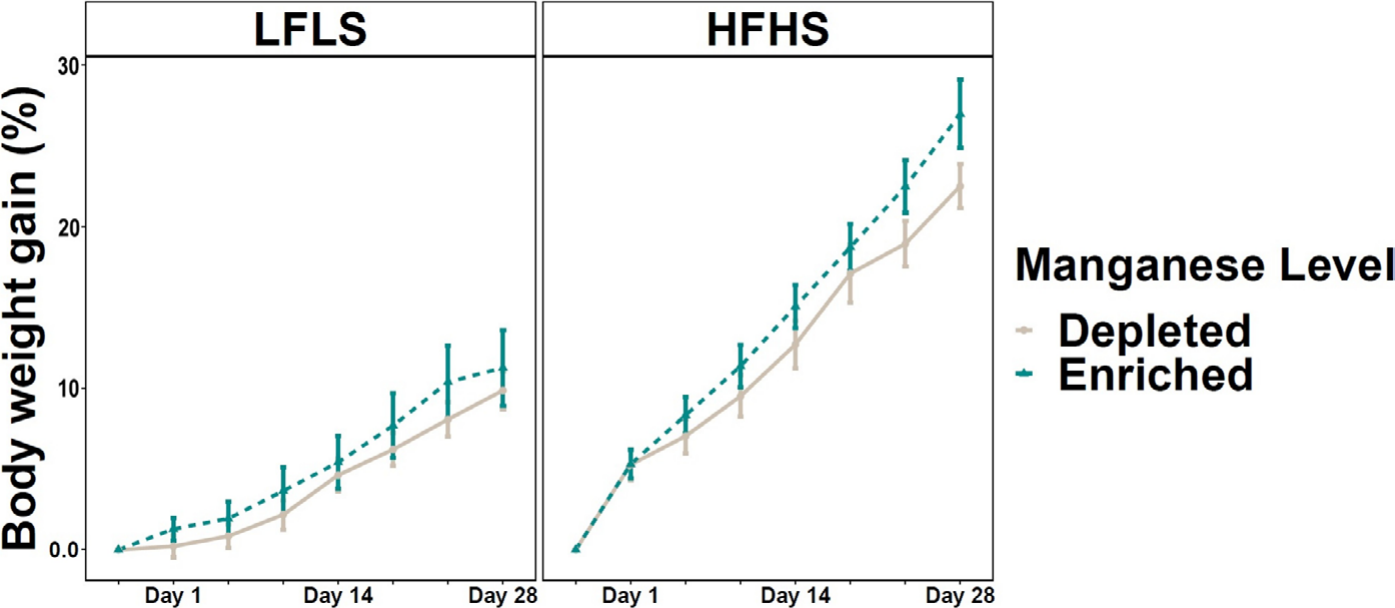
Weight gain in mice fed Mn‐enriched and Mn‐depleted LFLS or HFHS diets. Groups of 12 mice (6F/6M) were fed Mn‐enriched and/or Mn‐depleted diets combined with a LFLS or HFHS diet for 28 days. Generalized linear regression models were used to identify the effects of time or Mn and interactions with diet formulation. Data are expressed as mean ± SEM (*n* = 12).

Variations in dietary Mn intake showed no clear effect on the weight gain of the mice after 28 days. However, as expected, mice fed HFHS diets showed an increase in weight in comparison with LFLS (Figure 1). As for sex differences, male mice showed a greater weight gain than females on both Mn‐enriched and Mn‐depleted diets (Figure S1). These results suggest that Mn has a limited impact on weight gain in mice for the period of treatment.

### 3.2 Dietary Manganese Impacts on Circulatory and Caecal Levels of *N*‐Acylethanolamines

**FIGURE 2:**
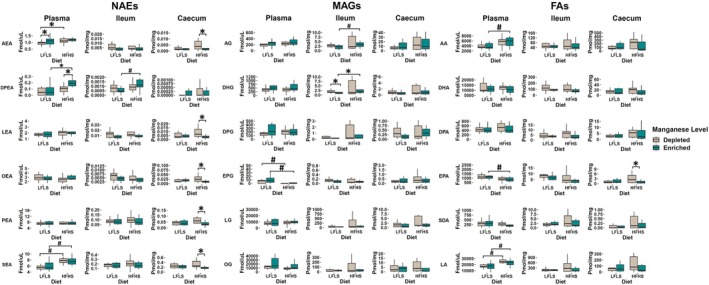
Diet and Mn modulation of endocannabinoidome mediators and some of their corresponding fatty acids. Boxplot representation of the response to Mn‐depleted and Mn‐enriched LFLS or HFHS diets of eCBome mediators: (Left) *N*‐acylethanolamines (NAEs), (Center) 2‐monoacylglycerols (2‐MAGs), and (Right) long chain *ω*‐6 and *ω*‐3 polyunsaturated fatty acids (FAs). Data are expressed as the mean ± SEM (*n* = 12). *p* values of linear contrast analysis are detailed when significant (*p* < 0.05) using contrast tests between enriched and depleted Mn levels, LFLS, and HFHS formulations and the combination between Mn levels and formulations. The star “*” symbol was used to show the effect of Mn alone or interaction with LFLS or HFHS. The numeral “#” symbol was used to denote the effect of only LFLS or HFHS. The samples were analyzed at Day 28 of the study.

We analyzed the concentrations of eCBome mediators (NAEs, MAGs) and the corresponding free fatty acids (FAs) in plasma, ileum, and caecum (Figure 2). The eCBome response was found to be different between plasma and the two intestinal segments studied. In plasma, we found that the *N*‐acylethanolamine DPEA showed a statistically significant increase associated with Mn enrichment in combination with the HFHS diet (*p* = 0.001). On the other hand, circulating levels of AEA were reduced in mice fed with Mn‐depleted LFLS diets. SEA was solely modulated by diet, as it was higher with HFHS than with LFLS diets. The most striking effect was observed at the intestinal level, where the Mn depletion with the HFHS diet led to a significant increase in several *N*‐acylethanolamines at the caecal level, namely AEA, LEA, OEA, PEA, and SEA. This effect was not significant in the ileum. Mn intake had a limited impact on 2‐MAGs, as only 2‐DHG showed a reduction associated with the Mn‐enriched LFLS diet in the ileum (*p* = 0.04). The free fatty acids were mostly affected by diet composition, except for eicosapentaenoic acid (EPA) in the caecum, which showed an increase associated with the Mn‐depleted HFHS diet (*p* = 0.02). Taken together, these results indicate that Mn depletion influences the intestinal levels of *N*‐acylethanolamines.

### 3.3 Dietary Manganese Affects the Levels of Intestinal Fatty Acid Amides and of Prostaglandins

**FIGURE 3:**
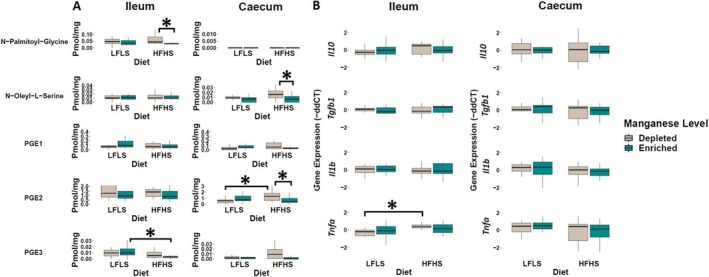
Response of some fatty acid amides and prostaglandins, and mRNA gene expression of immune response markers in Mn‐depleted and Mn‐enriched LFLS or HFHS diets in the intestine. (A) Boxplot of fatty acid amides and prostaglandins in ileum and caecum. (B) mRNA expression of immune response as fold change (FC) calculated using the ΔΔ*C*_*T*_ method in Mn‐depleted and Mn‐enriched LFLS or HFHS diets in the intestine, ileum, and caecum. Data are expressed as the mean ± SEM (*n* = 12). *p* values of linear contrast analysis are detailed when significant (*p* < 0.05) using contrast tests between enriched and depleted Mn levels, LFLS and HFHS formulations and the combination between Mn levels and formulations. Gene expression was normalized to *Hprt*. The star “*” symbol was used to show the effect of Mn alone or interaction with LFLS or HFHS. The samples were analyzed at Day 28 of the study.

In addition to endocannabinoid congeners and FAs, we also quantified other lipid mediators and eicosanoids that could respond differentially to changes in dietary Mn (Figure 3A). We found that Mn‐enriched HFHS diets reduced the concentrations of *N*‐palmitoyl‐glycine in the ileum (*p* = 0.05). This molecule was below the limit of detection in the caecum. In parallel, elevation of *N*‐oleoyl‐L‐serine with the Mn‐depleted HFHS diet was observed in the caecum (*p =* 0.03). Concerning prostaglandins, Mn‐depletion with HFHS diet was associated with the elevation of PGE_2_ in the caecum (*p =* 0.02), while the interaction of Mn‐enriched diets with LFLS favored the increase of PGE_3_ compared to HFHS diet. Dietary Mn variations did not modulate the expression levels of genes involved in inflammation, except for *Tnfa* in the ileum, which was downregulated in LFLS diet with depleted Mn (Figure 3B). Overall, the effects of Mn on inflammation‐related variables were limited in this study compared to other microelements such as Selenium (Se) [20].

### 3.4 Dietary Manganese Impacts Specific Taxa of the Gut Microbiome

**FIGURE 4:**
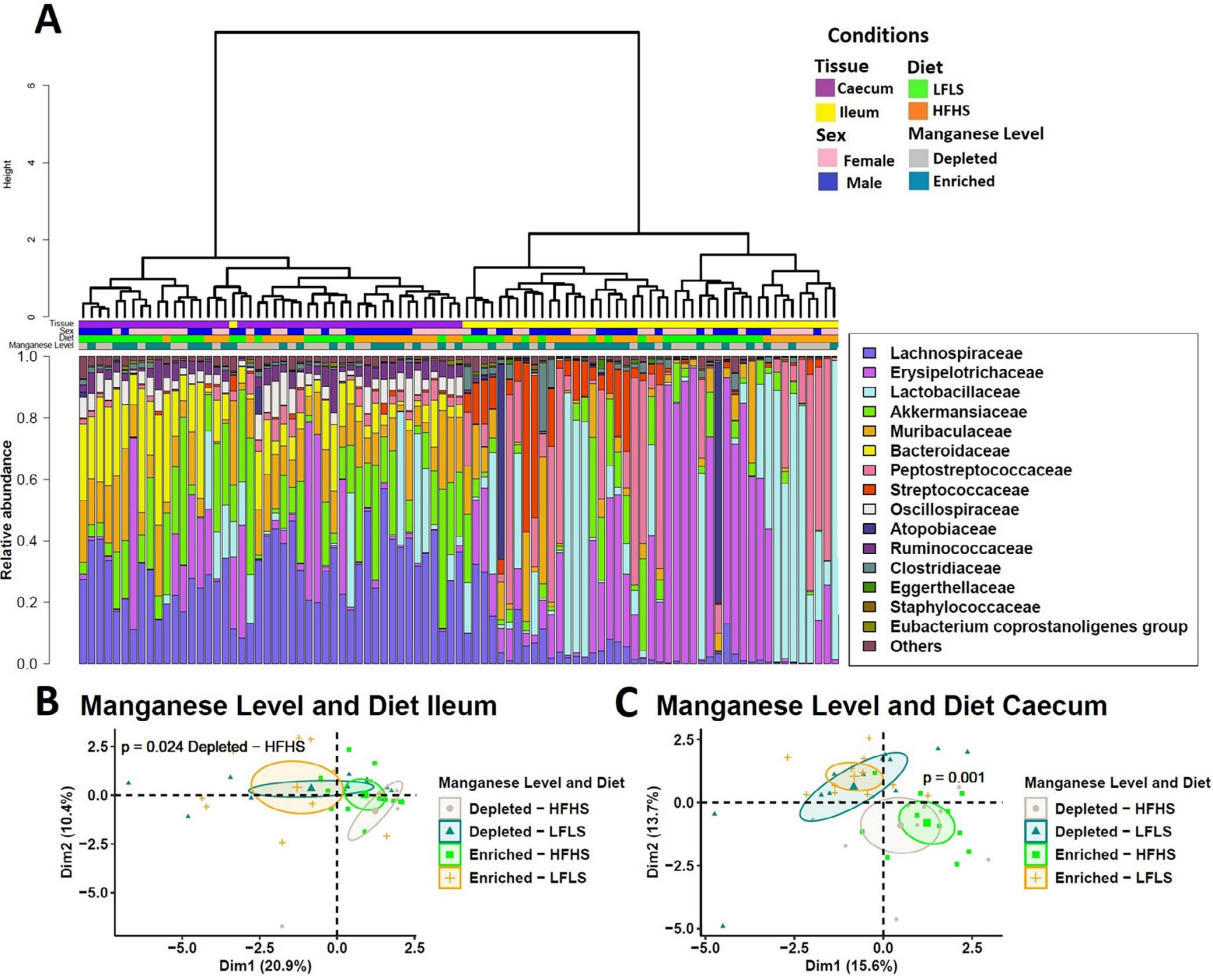
Intestinal microbiota composition in response to Mn‐enriched and Mn‐depleted LFLS or HFHS diets. (A) Relative bacterial abundance at the family level in response to Mn‐depleted and Mn‐enriched LFLS or HFHS diets in ileum, and caecum. Families representing < 1% of total bacterial abundance were aggregated. Dendrogram showing hierarchical clustering based on Canberra distance between samples is showed and determines the sample order. The corresponding annotations for tissue, sex, diet, and Mn level are displayed. (B) Principal component analysis (PCA) shows the impact of depleted/enriched concentrations of Mn with High and Low‐Fat Sucrose diets on gut microbiota composition of (B) the ileum and (C) the caecum. PERMANOVA indicates significance of microbiota composition differences between the dietary conditions. The samples are at Day 28 of the study.

**FIGURE 5:**
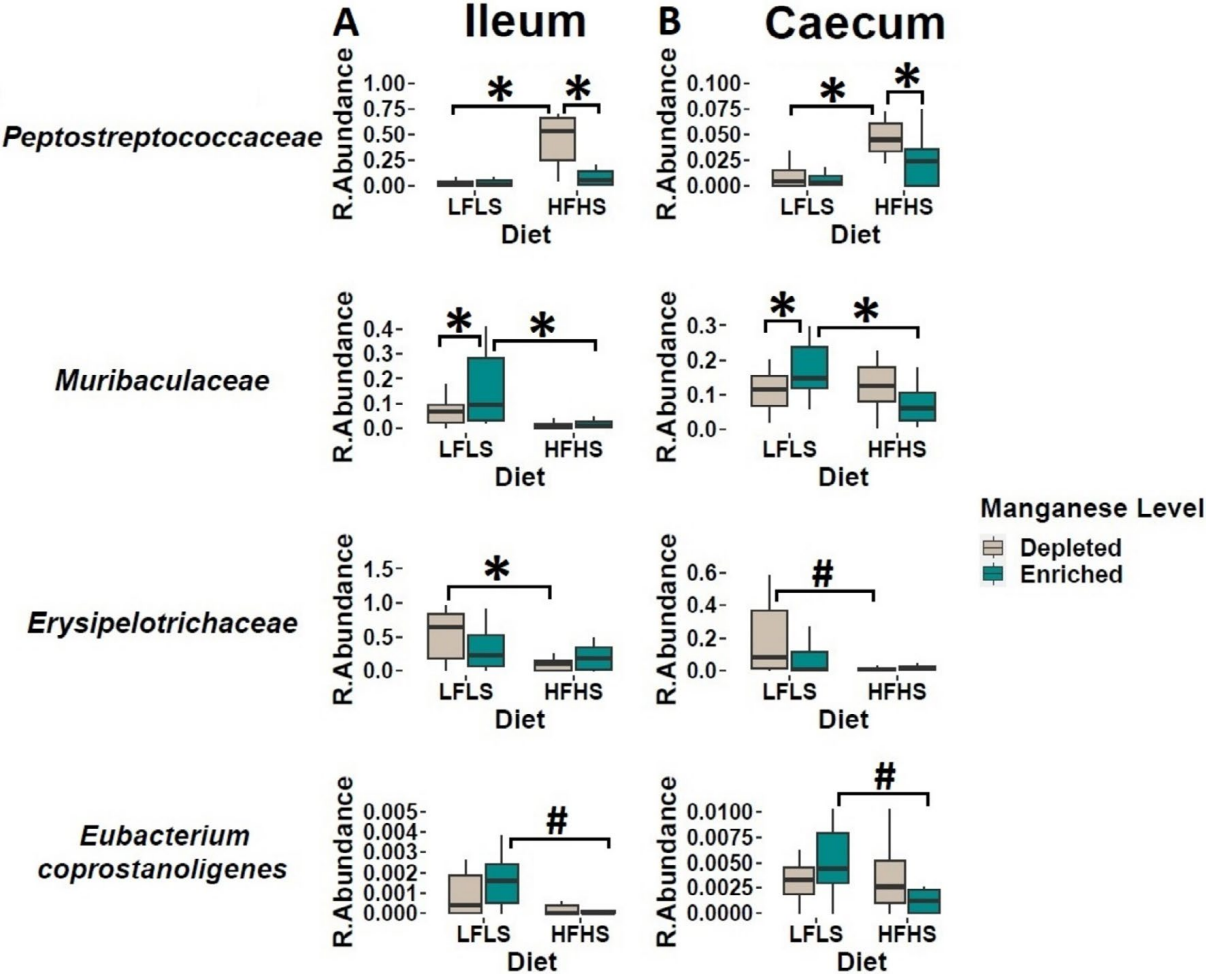
Effect of Mn‐depleted and Mn‐enriched LFLS or HFHS diets on the relative abundance of bacteria at the family rank in either the ileum or caecum. Effect of Mn in interaction with LFLS and HFHS formulations on intestinal microbial families. Ileum (A) and caecum (B). Data are expressed as the mean ± SEM (*n* = 12). *p* values of linear contrast analysis are detailed in the bottom when significant (*p* < 0.05) using contrast tests between enriched and depleted Mn levels, LFLS and HFHS formulations and the combination between Mn levels and formulations. The star “*” symbol was used to show the effect of Mn alone or interaction with LFLS or HFHS. The numeral “#” symbol was used to denote the effect of only LFLS or HFHS. The samples are at Day 28 of the study.

We investigated whether specific gut microbial families were differentially sensitive to dietary variations of Mn and whether this association remained independent of dietary fat and sucrose intake. As observed previously [20], intestinal microbiota composition showed a remarkable differentiation between the segments of the intestine (*p* < 0.01, PERMANOVA), with discernible impact of diet composition in both segments, although more pronounced in the caecum than ileum, and no major effect of Mn on sample clustering (Figure 4). Three intestinal microbial families responded directly to Mn intake or to the interaction between Mn and diet content. *Peptostreptococcaceae* showed a decrease in relative abundance under Mn‐enriched diets in combination with HFHS in both ileum and caecum intestinal segments (*p* < 0.01) (Figure 5A,B). Instead, *Muribaculaceae* showed a statistically significant increase with Mn‐enriched diets in combination with LFLS in both ileum and caecum. *Erysipelotrichaceae* exhibited an increase in its relative abundance with Mn‐depleted diets in ileum (*p* < 0.01) (Figure 5A). Finally, *Eubacterium coprostanoligenes* showed an increase in its relative abundance associated solely with LFLS in both tissues (*p* = 0.03). Taken together, specific microbial taxa responded to dietary variations in Mn, while others responded to changes in an exclusively diet‐dependent manner and based on the intestinal segment.

### 3.5 Intestinal 2‐Monoacylglycerols (2‐MAGs) and Specific Microbial Families Were Modulated in a Sex‐Dependent Manner

**FIGURE 6:**
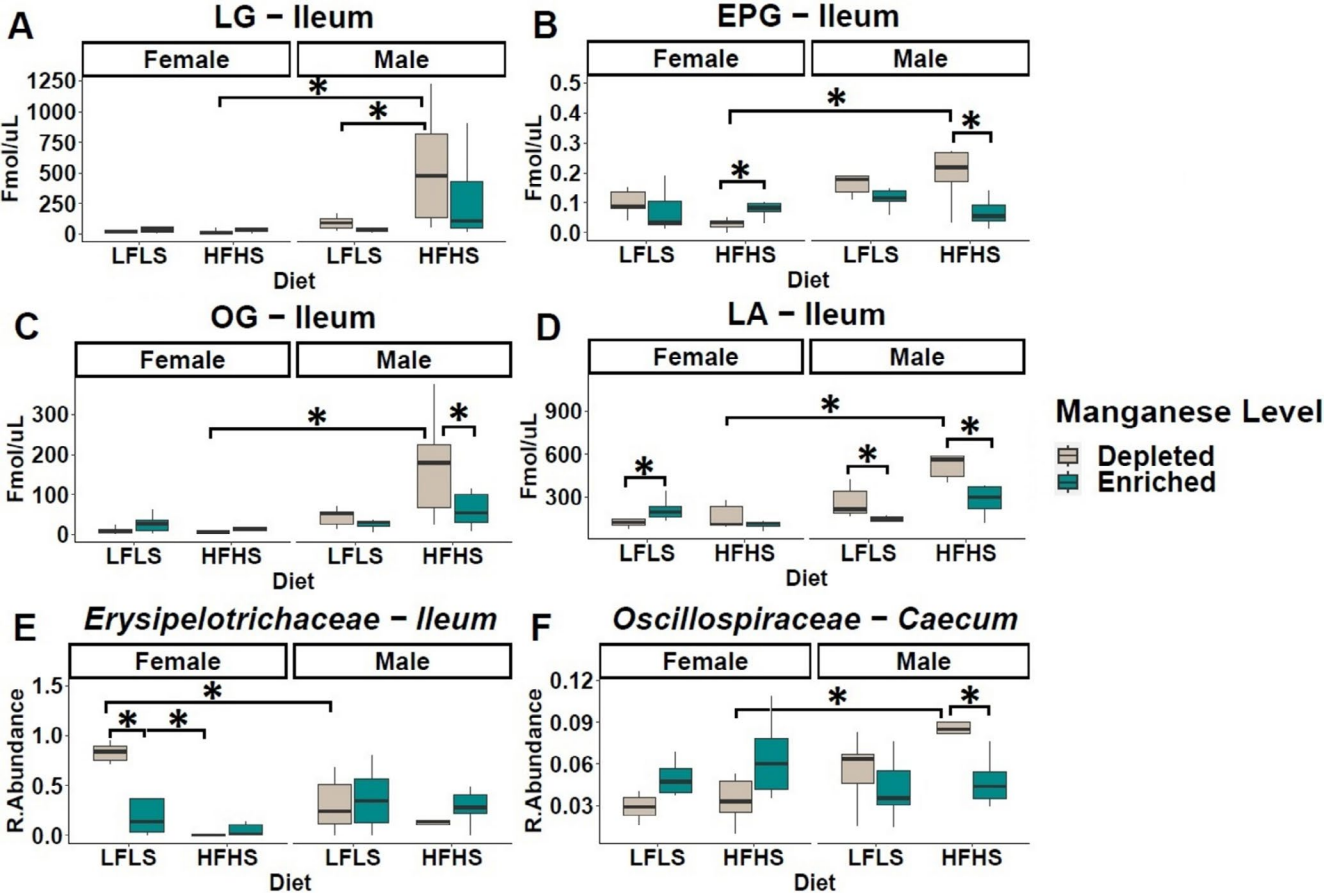
Manganese influences in a sex‐dependent manner intestinal 2‐monoacylglycerols (MAGs), and specific intestinal microbial families. (A–C) Boxplot of the MAGs in ileum, (D) long chain ω‐6 fatty acid (LA), (E, F) and microbial families in the ileum and caecum. Data are expressed as the mean ± SEM (*n* = 48). *p* values of linear contrast analysis are marked with a star “*” when significant (*p* < 0.05) using contrast test between enriched and depleted Mn levels, LFLS and HFHS formulations, the combination between Mn levels and formulations, and the sex of the animal. The samples analyzed and showed are at Day 28 of the study.

Beyond the impact of dietary Mn and the interaction with dietary fat/sucrose formulations, we also considered the role of sex on the effect of Mn on eCBome mediators, intestinal immune response, and intestinal microbial taxa. We found that, in the ileum, the levels of 2‐EPG, 2‐OG, and 2‐LG showed an increase in association with Mn depletion and the HFHS diet only in males (*p* = 0.001) (Figure 6A–C). The only FA that responded to Mn interaction with the sex of mice was LA, which exhibited an elevation in males under the Mn‐depleted diets in combination with HFHS (Figure 6D). Concomitantly, microbial families such as *Erysipelotrichaceae* exhibited an increase in their relative abundance in the ileum that was associated with the Mn‐depleted LFLS diet in female mice (*p* < 0.01). *Oscillospiraceae* showed a contrasting response in the caecum between males and females, as it was higher in males with the Mn‐depleted HFHS diet compared to the same condition in females and to the Mn‐enriched HFHS diet in males (*p* = 0.03) (Figure 6E,F). Overall, the interaction between dietary Mn levels and sex allowed the modulation of intestinal 2‐MAGs and specific intestinal microbial families.

### 3.6 Ileal Microbiota and Lipid Mediator Interactions in Response to Dietary Variations of Mn

**FIGURE 7:**
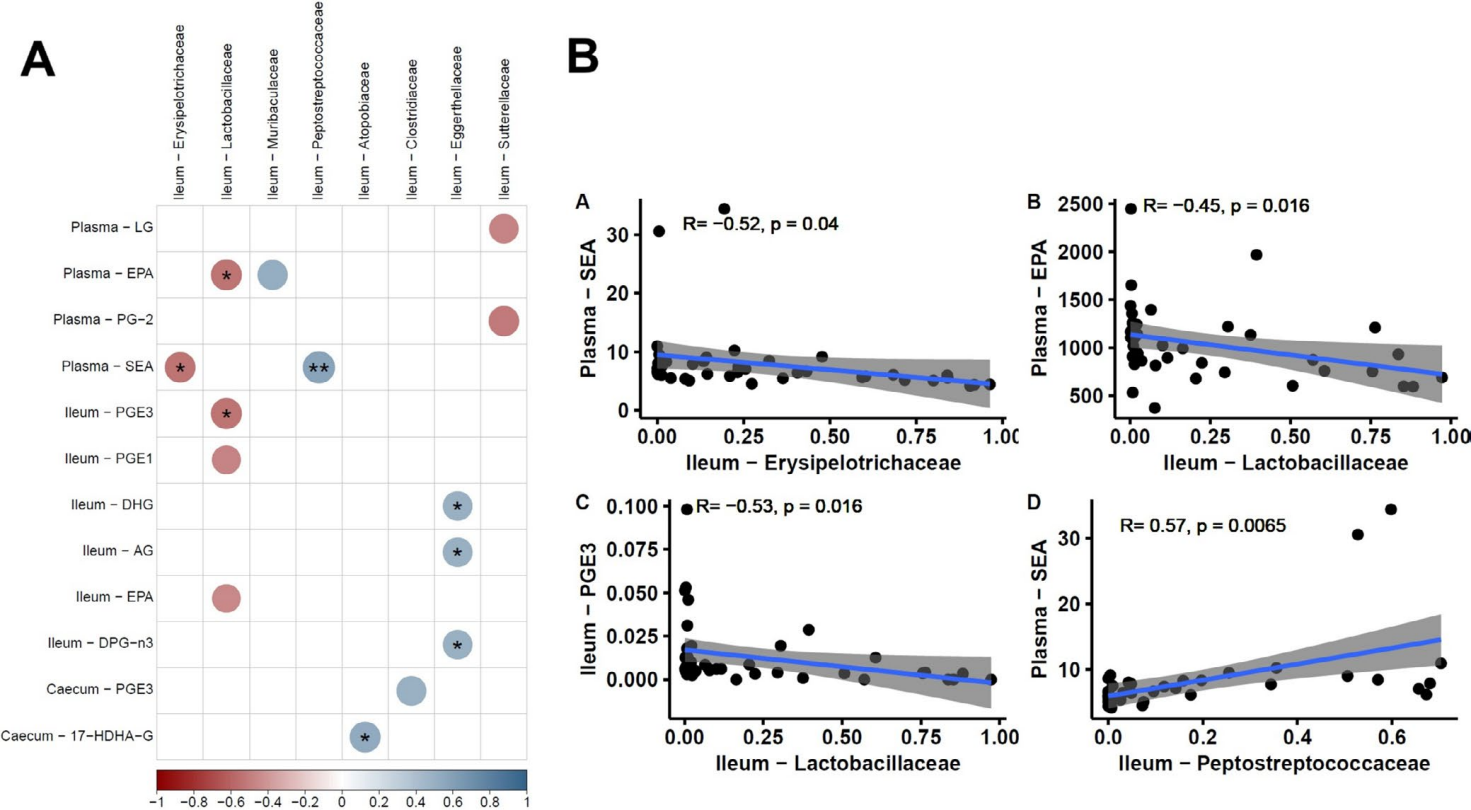
Correlation between ileum microbiota composition and lipid mediators in mice fed Mn‐depleted and ‐enriched LFLS or HFHS diets. (A) Heatmap illustrates Spearman correlation coefficients between the relative abundance of each microbial family and the level of eCBome mediators in plasma, ileum, and caecum. Significance was set at *p* < 0.05 (*) and *p* < 0.01 (**). (B) Scatterplots refer to the FDR‐adjusted Spearman rho correlations *n* = 47.

We investigated interactions between the eCBome and the microbiome using Spearman correlations. The heatmap in Figure 7 illustrates the correlation coefficients of microbiota families with eCBome mediators in both plasma and ileum. The relative abundance of *Erysipelotrichaceae* in the ileum was negatively associated with circulating levels of SEA (*r* = −0.28, false‐discovery rate [FDR] *p* = 0.071, *n* = 47). Similarly, the microbial family *Lactobacillaceae* was negatively correlated with circulatory EPA, intestinal EPA, and PGE_3_ (−0.34 < *r* < −0.32 = −0.28, false‐discovery rate [FDR] *p* < 0.05, *n* = 47), and the microbial family *Peptostreptococcaceae* was positively correlated with circulatory SEA (*r* = 0.5, false‐discovery rate [FDR] *p* < 0.05, *n* = 47). Taken together, these data suggest that intestinal microbial families, particularly in the ileum, are associated with circulatory changes of specific lipid mediators.

## 4 Discussion

In the present study, we examined the effect of Mn depletion (0.1 mg/kg) and enrichment (20 mg/kg), in combination with macronutrients (LFLS or HFHS), on eCBome and gut microbiota in C57BL/6J mice. In contrast with several studies that used diets with < 0.1 Mn/kg to describe the underlying metabolic defects related to severe Mn deficiency [35], we did not target severe dietary Mn depletion, or the occurrence of brain injuries in the Mn‐enriched diets [36]. Our model was not designed to produce a negative impact on mice associated with a drastic reduction in Mn intake or severe Mn overload. Similarly, our aim was not to produce systemic and tissue inflammation. However, previous studies have shown that 4 weeks of obesogenic diet in mice is sufficient to modify the inflammatory phenotype and to provoke changes in the intestinal microbiota [20, 27]. This study should therefore be considered a short‐term study, since it covered a period of 4 weeks, which characterizes the rapid adaptability of the host to dietary changes. It is important to note that we observed no statistically significant differences in weight gain associated with Mn depletion or enrichment with the diets used, which is consistent with results that have been previously reported [37]. However, Mn intake has been reported to have beneficial effects in reversing metabolic dysfunction [38], and to be important in regulating blood lipid metabolism [39], which corresponds to the results we obtained here. We show that the intake of dietary Mn is associated with changes in the circulating and intestinal eCBome. Circulatory levels of DPEA showed a statistically significant increase associated with Mn enrichment in combination with HFHS diet, while AEA was reduced with depleted Mn in LFLS diet. These mediators, which showed higher concentration with the HFHS diet, were also found to be more abundant in individuals with chronic systemic inflammation [40], or, in the case of AEA, it has been reported to exert effects on inflammation in obesity [41, 42, 43]. Thus, dietary variations in Mn could constitute in responses aimed at counteracting or enhancing inflammation. Regulation of the eCBome varies depending on intestinal segment, metabolic and inflammatory status [44]. In the caecum, dietary Mn depletion with HFHS diet led to an increase in *N*‐acylethanolamines, especially AEA, LEA, OEA, PEA, and SEA. In the ileum, NAEs were not modulated. The link between increased intestinal levels of NAEs and dietary Mn depletion might be explained by the fact that Mn deficiency may affect the expression of genes involved in the synthesis and degradation of NAEs, which may contribute to higher levels of NAEs, since it has previously been reported that Mn deficiency can alter enzyme activity involved in lipid and carbohydrate metabolism [45].

Nevertheless, the Mn‐enrichment may influence the development of obesity through selective modulation of specific endocannabinoid mediators in the gut, particularly NAEs, such as PEA and OEA, are known to exert anti‐inflammatory and anorexigenic effects through activation of PPAR‐α and inhibition of feeding behavior. Here, we demonstrated that Mn‐enrichment has been associated with reduced NAEs levels in the caecum, which could decrease its protective metabolic functions and contribute to increased appetite and adiposity [30]. By contrast, Mn supplementation has been observed to increase levels of circulatory levels of DPEA. Although DPEA may exert anti‐inflammatory effects through PPAR‐γ [46], its metabolic function appears to be context‐dependent, and its accumulation under Mn‐enriched conditions could reflect a compensatory response to Mn‐induced changes in lipid signaling or microbial metabolism [47, 48]. This switch‐from metabolically protective NAEs to increased DPEA‐may signal an altered lipid‐mediated environment that contributes to dysregulation of the gut‐brain axis and promotes the metabolic imbalance characteristic of obesity.

Previous studies have shown that Mn metabolism was related to lipid peroxidation [49, 50, 51]. Other studies have shown that Mn participates in lipid metabolism through lipid synthesis [52]. Likewise, we found that Mn depletion selectively affected ileal 2‐MAGs, including 2‐EPG, 2‐LG and 2‐OG, in a sex‐dependent manner. Whereas C57BL/6J mice typically reach sexual maturity at approximately 8 weeks of age [53], our study began at 6 weeks and extended over a 4‐week period. Consequently, all animals reached sexual maturity during the experimental period. These results are consistent with previous reports on sex‐based differences in basal eCBome mediator levels and may underlie in part the sex‐specific response to some nutritional interventions [26].

Endocannabinoids are derivatives of arachidonic acid (AA) esterified in the plasma membrane. AA is a polyunsaturated fatty acid (PUFA) released in large amounts by macrophages [54] and neutrophils [55] from membrane phospholipids during the inflammatory burst. It is then metabolized to potent signaling molecules, including prostaglandins, isoprostanes, leukotrienes, and hydroxytetraenoic acids [56, 57]. *N*‐acylamides have pleiotropic signaling functions in physiology, including in cardiovascular function, metabolic homeostasis, memory, cognition, pain, motor control, and others [58]. Besides, *N*‐acylamides are an emerging family of endogenous lipid signaling molecules with structural similarities to eCBs that can also bind eCBome receptors [59]. Indeed, *N*‐acylamides were shown to prevent the cytokine‐induced increase in gut permeability in an animal model [60]. Mn may be involved in the increased production of these endogenous fatty acid compounds since we found that Mn‐enriched diets modulated ileal *N*‐palmitoyl‐glycine and caecal *N*‐oleoyl‐L‐serine. Concomitant with the modulation of caecal *N*‐oleoyl‐L‐serine, we also observed an increase of PGE_2_ with the Mn‐depleted HFHS diet, which is indicative of the role that dietary amounts of Mn would play in the modulation of intestinal inflammatory mediators with a HFHS diet. Prostaglandins are produced from AA, and their altered metabolism affects the production of reactive oxygen species [61]. Besides, prostaglandins and the endogenous long‐chain fatty acid amides studied here represent relevant inflammation‐related lipid mediators [62]. Taken together, these results suggest that dietary Mn enrichment could influence intestinal *N*‐acylamide (as well as NAEs and 2‐MAGs in male mice) and prostaglandin production, thereby affecting the intestinal inflammatory response.

A limitation of this study is the absence of functional metabolic measurements such as glucose tolerance tests or indirect calorimetry, as well as the lack of protein‐level quantification of inflammatory markers or circulating cytokines, which would provide a more complete picture of the systemic metabolic and immune status. However, to better gain insight into the local inflammatory environment, we assessed gene expression levels of key proinflammatory cytokines (*Il1b* and *Tnfα*) and anti‐inflammatory markers (*Il10* and *Tgfb1*) and prostaglandins in intestinal tissues. These transcriptional profiles and lipidomic quantifications provide relevant information on the immune responses of the intestinal tract associated with the dietary Mn‐intervention, although mRNA expression might not directly reflect protein abundance or activity. In addition, it is possible that the number of animals used in this study might not provide enough statistical power to detect small modulations of some bacteria or metabolites. Future studies incorporating validation at the level of proteins and systemic markers will be important to strengthen the mechanistic understanding of the observed effects.

Dietary variations in Mn modulated specific bacterial families in both the ileum and caecum. Mn deficiency, particularly with the HFHS diet, was associated with the increase of *Peptostreptococcaceae*, while Mn enrichment favored *Muribaculaceae* with the LFLS diet, suggesting that specific bacterial families will take advantage of increased dietary Mn, while others are sensitive to its reduction. As an essential trace mineral, Mn can be absorbed and utilized by many bacterial species [63]. In the gastrointestinal tract, Mn is normally maintained at low levels and bacteria that require Mn for growth, for example *Lactobacillus*, are inhibited due to limited Mn availability [64]. In all life forms, minerals are required for the enzymatic function of more than 36% of the proteins of all enzyme classes [65]. Considering this essential requirement of minerals for cellular function in both vertebrate hosts and bacterial pathogens, it is not surprising that, in response to bacterial infections, vertebrates undergo drastic alterations in mineral metabolism. Besides, many strategies have been developed by vertebrates to deprive bacteria of these nutritional micronutrients through a process referred to as nutritional immunity [66]. Through Mn sequestration, the host might express proteins such as calprotectin in the mucosa, which can directly bind Mn to reduce its availability and thus prevent microbial growth, leading to nutritional immunity [67]. Therefore, dietary Mn depletion could reduce Mn in the gastrointestinal tract, which could favor bacteria such as *Peptostreptococcaceae*. Some microbial species have been reported to use siderophores to scavenge Fe as a survival mechanism when availability of Fe is limited [68, 69]. Interestingly, when stratified for the sex of the animals, the significant increase in *Erysipelotrichaceae* with the Mn‐depleted LFLS diet remained significant in female mice but not in males, suggesting a sex‐specific response. Concomitantly, in male mice, Mn‐depleted diets were associated with a significant increase in the relative abundance of *Oscillospiraceae* under the HFHS diet. These results are coherent with previous reports showing that Mn exposure has sex‐specific effects on intestinal bacteria [25], and potentially indicate that the gut bacteria of males and females could exhibit different responses to Mn and potentially diverge in functions relatively to the metabolism of this mineral. Yet, the mechanism underlying the sex‐dependent response of the gut microbiome in the present study remains unclear. However, sex hormones, such as estrogen and testosterone are highly involved in both Mn metabolism and the modulation of the eCBome. Estrogens have been shown to regulate Mn transporters [70], potentially altering Mn uptake and tissue distribution in a sex‐dependent manner. This may contribute to the observed differences in Mn response between males and females. Similarly, sex hormones modulate the eCBome as estrogens tend to down‐regulate levels of endocannabinoids such as anandamide (AEA) and 2‐arachidonoylglycerol (2‐AG) [71], while testosterone may enhance their signaling [72, 73], leading to sex‐specific differences in CB_1_ receptor activation. These hormonal effects impact not only appetite and fat storage, but also the gut microbiota, which in turn may influence both Mn bioavailability and eCBome lipid mediator profiles. Consequently, the interplay between sex hormones, Mn status and eCBome may contribute to differential susceptibility to obesity and metabolic diseases in males and females. In addition, the expression of genes related to Mn metabolism differs between males and females [74]. It has also previously been shown that Mn interacts with nutrients, such as iron (Fe), zinc (Zn), and copper (Cu), differently in males and females [75]. Our findings confirm that micronutrients such as Mn exert profound effects on intestinal populations. Additionally, we found that *Erysipelotrichaceae* and *Lactobacillaceae* were negatively correlated with circulatory SEA and EPA respectively, whereas *Peptostreptococcaceae* was positively correlated with circulatory SEA. In humans, *Peptostreptococcaceae* have also been reported to correlate with blood levels of NAEs [76]. Limited data is available on the relationship between *Peptostreptococcaceae* and metabolism. A possible link between NAEs and this group of anaerobes could be their fermentative metabolism [77], as omega‐3 PUFA‐derived NAEs could be hydrolyzed by microbial lipases with release of free fatty acids and fermentation of non‐fatty acid components [78].

## 5 Conclusions

Overall, our results emphasize the fundamental structuring effect of Mn and diet on phenotype. On one side, Mn intake modulates the intestinal eCBome response in interaction with the macronutrient diet composition. Specifically, Mn enrichment reduced the levels of caecal NAEs. Additionally, we found a strong sex‐dependent Mn‐induced response affecting ileal 2‐MAG levels only in male mice, pointing to the differential role that this trace element may have for males and females. On the other hand, dietary Mn enrichment was associated with reduction of intestinal fatty acid amide and prostaglandin levels, in agreement with the role of this transition mineral in the modulation of intestinal inflammation and metabolic health. Concomitantly, variations in dietary Mn concentrations, particularly its depletion, favored the relative abundance of *Peptostreptococcacea* and specific microbial families responded to the effect of Mn in a sex‐dependent manner. The results presented here should foster further studies exploring the molecular basis of how micronutrients impact the gut microbiome‐eCBome axis, particularly in relation to Mn deficiency or supplementation.

## Author Contributions

**Fredy Alexander Guevara Agudelo**, **Nadine Leblanc**, **Vincenzo Di Marzo**, and **Frédéric Raymond:** conceptualization. **Fredy Alexander Guevara Agudelo**, **Nadine Leblanc**, **Gabrielle St‐Arnaud**, and **Fadil Dahhani:** methodology and data acquisition. **Fredy Alexander Guevara Agudelo**, **Isabelle Bourdeau‐Julien**, **Nicolas Flamand**, **Vincenzo Di Marzo**, and **Frédéric Raymond:** formal analysis. **Fredy Alexander Guevara Agudelo** and **Frédéric Raymond:** data curation. **Fredy Alexander Guevara Agudelo**, **Vincenzo Di Marzo**, and **Frédéric Raymond:** writing – original draft. **Fredy Alexander Guevara Agudelo**, **Isabelle Bourdeau‐Julien**, **Nicolas Flamand**, **Gabrielle St‐Arnaud**, **Nicolas Flamand**, **Alain Veilleux**, **Vincenzo Di Marzo**, and **Frédéric Raymond:** writing – review and editing. **Vincenzo Di Marzo** and **Frédéric Raymond:** supervision. **Vincenzo Di Marzo** and **Frédéric Raymond:** funding acquisition.

## Conflicts of Interest

The authors declare no conflicts of interest.

## Supporting information


**Figure S1.** Weight gain in Female and Male mice fed with fed Mn‐enriched and Mn‐depleted LFLS or HFHS diets. Groups of 12 mice (6F/6M) were fed Mn‐enriched and/or Mn‐depleted diets for 28 days. Generalized linear regression models were used to identify the effects of time or Mn and interactions. Data are expressed as mean ± SEM (*n* = 6).

## Data Availability

Raw sequences were deposited to SRA under accession PRJNA1005664. The primers used are available at Thermofisher website: (https://www.thermofisher.com/ca/en/home/life‐science/pcr/real‐time‐pcr/real‐time‐pcr‐assays.html) with the TaqMan assay IDs as follows: *Hprt* (Mm03024075_m1), *Il10* (Mm01288386_m1), *Tgfb1* (Mm01178820_m1), *Il1b* (Mm00434228_m1), and *Tnfa* (Mm00443258_m1).
