# Supplementary figures and images for: Dietary Manganese Modulates Microbiota and Intestinal N‐Acylethanolamines in a Sex‐Specific Manner in Mice With Diet‐Induced Obesity
Source: FASEB J. 2025 Jun 26;39(13):e70763. doi: 10.1096/fj.202500934R (PMC12199783; doi:10.1096/fj.202500934R)

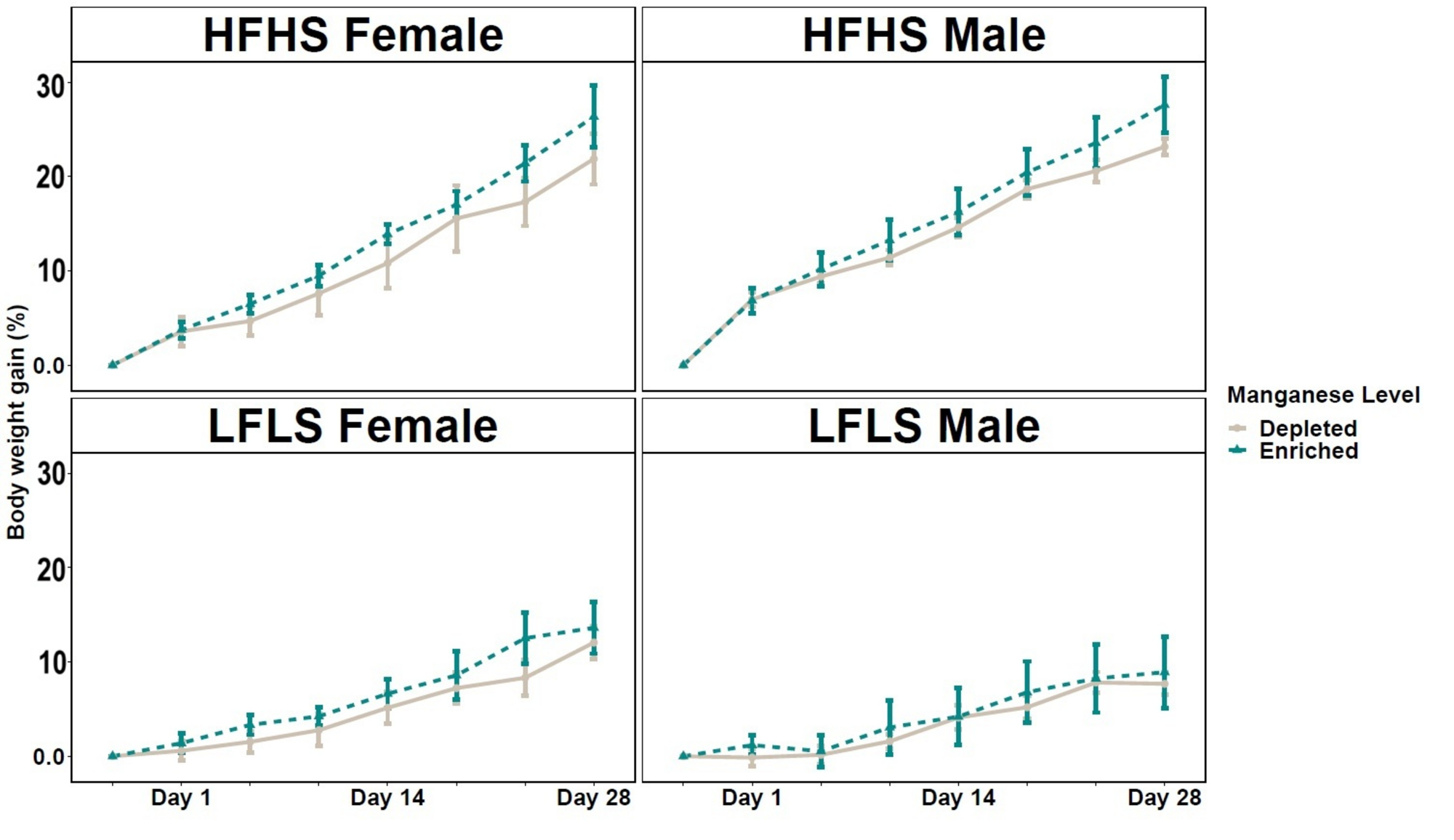

Supplement: Supplementary file 1 — Figure S1. Weight gain in Female and Male mice fed with fed Mn‐enriched and Mn‐depleted LFLS or HFHS diets. Groups of 12 mice (6F/6M) were fed Mn‐enriched and/or Mn‐depleted diets for 28 days. Generalized linear regression models were used to identify the effects of time or Mn and interactions. Data are expressed as mean ± SEM (n = 6). [file FSB2-39-e70763-s001.tif]
